# Supplementary material for: Headlines win elections: Mere exposure to fictitious news media alters voting behavior
Source: PLoS One. 2023 Aug 1;18(8):e0289341. doi: 10.1371/journal.pone.0289341 (PMC10393126; doi:10.1371/journal.pone.0289341)
Supplement: S1 Text — (DOCX) [file pone.0289341.s001.docx]

**Supplementary Analyses**

*Experiment 1*

Figure S2A compares the main analyses to the three analyses of subsets of the data; detailed statistics are reported in Table S1. A stable preference for the frequent name was evident in all analyses, *p*s < .001, *BF*_01_ ≥ 16610, with effect size estimates ranging from w = 0.59 (subset: Name not mentioned) to w = 0.65 (Valence not mentioned).

This pattern of results did not depend on self-reported interest in the headlines, β_logit_ = -0.007, *p* = .489, attention allocated to the newspaper content, β_logit_ = 0.002, *p* = .880, or potential suspicions about the role of this content for later stages of the experiment, β_logit_ = 0.004, *p* = .709

*Experiment 2*

Figure S2B compares the main analyses to the three analyses of subsets of the data; detailed statistics are reported in Table S2. None of the tests was significant, *p*s > .111 (*p*_exact_ ≥ .137) though Bayes Factors suggested that the pattern of results was ambiguous, 0.425 ≤ *BF*_10_ ≤ 0.831, underlining the small effect sizes towards a preference for the infrequent name with an estimated w ranging between 0.11 (Name not mentioned) and 0.17 (Main analysis).

As for the first experiment, this pattern of results did not depend on self-reported interest in the headlines, β_logit_ = 0.003, *p* = .756, attention allocated to the newspaper content, β_logit_ = 0.004, *p* = .522, or potential suspicions about the role of this content for later stages of the experiment, β_logit_ = 0.006, *p* = .383.

*Experiment 3*

Figure S2C compares the main analyses to the three analyses of subsets of the data; detailed statistics are reported in Table S3. A stable preference for the frequent name was evident in all analyses, *p*s ≤ .007, with effect size estimates ranging from w = 0.25 (Name not mentioned) to w = 0.29 (Full sets only; Valence not mentioned).

The pattern was again independent of the participants’ self-reported engagement with the newspaper content; interest: β_logit_ = -0.003, *p* = .616; attention: β_logit_ = 0.007, *p* = .283, suspicions: β_logit_ = 0.0003, *p* = .962.

In the final candidate evaluation, the candidate that had appeared frequently in the newspaper task was rated as more active than the competing candidate (77.54 %VAS vs. 64.42 %VAS), *t*(138) = 5.09, *p* < .001, *d* = 0.43. The same held true for assertiveness (70.66 %VAS vs. 60.02 %VAS), *t*(138) = 4.21, *p* < .001, *d* = 0.36.

*Experiment 4*

Figure S2D compares the main analyses to the three analyses of subsets of the data; detailed statistics are reported in Table S4. A stable preference for the frequent name was evident in all analyses, *p*s < .001, with effect size estimates ranging from w = 0.36 (Name not mentioned) to w = 0.40 (Valence not mentioned).

Analyses of the debriefing questions suggested that participants showed a smaller bias to vote for the frequent name the less interested they were in the headlines (i.e., the more annoyed they were by this content), β_logit_ = -0.020, *p* = .006. Attention allocated to the newspaper content showed a non-significant trend towards larger preferences for the frequent name when this content was attended, β_logit_ = 0.011, *p* = .053, and the same was true for potential suspicions about the role of this content for later stages of the experiment, β_logit_ = 0.011, *p* = .061.

In the final candidate evaluation, the candidate that had appeared in the newspaper task was rated as more active than the competing candidate (74.54 %VAS vs. 59.34 %VAS), *t*(141) = 5.27, *p* < .001, *d* = 0.44. The same held true for assertiveness (70.43 %VAS vs. 57.10 %VAS), *t*(141) = 4.86, *p* < .001, *d* = 0.41.
