# Supplementary material for: Headlines win elections: Mere exposure to fictitious news media alters voting behavior
Source: PLoS One. 2023 Aug 1;18(8):e0289341. doi: 10.1371/journal.pone.0289341 (PMC10393126; doi:10.1371/journal.pone.0289341)
Supplement: S1 Fig — They were instructed to monitor the developments on the stock market because they could invest in one of the five companies at the end of the experiment to secure a bonus. The remaining content of the page was irrelevant for the main task. However, each headline contained either the name Smith or Jones and, across the 40 news pages, one of the names appeared frequently whereas the other name appeared infrequently. (DOCX) [file pone.0289341.s002.docx]

Fig. S1.


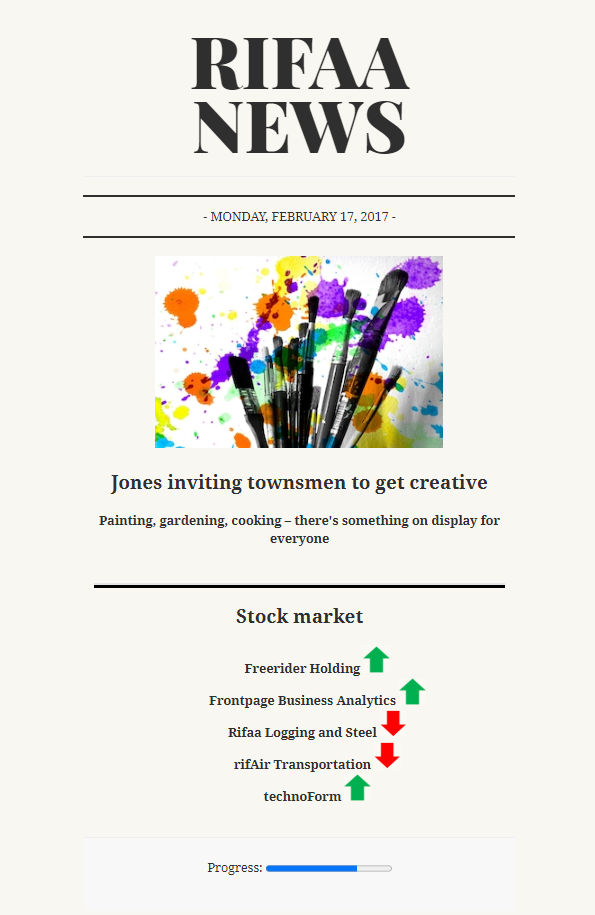


**Fig. S1.** Sample page of the newspaper task; a “Next” button appeared right below this display so that participants could browse through the pages in a self-paced manner. They were instructed to monitor the developments on the stock market because they could invest in one of the five companies at the end of the experiment to secure a bonus. The remaining content of the page was irrelevant for the main task. However, each headline contained either the name Smith or Jones and, across the 40 news pages, one of the names appeared frequently whereas the other name appeared infrequently.
