# Supplementary material for: Headlines win elections: Mere exposure to fictitious news media alters voting behavior
Source: PLoS One. 2023 Aug 1;18(8):e0289341. doi: 10.1371/journal.pone.0289341 (PMC10393126; doi:10.1371/journal.pone.0289341)
Supplement: S2 Fig — (DOCX) [file pone.0289341.s003.docx]

Fig. S2.

**Fig. S2.** Validation analyses for all four experiments including sample sizes for each subset of the data.
