# Supplementary material for: Headlines win elections: Mere exposure to fictitious news media alters voting behavior
Source: PLoS One. 2023 Aug 1;18(8):e0289341. doi: 10.1371/journal.pone.0289341 (PMC10393126; doi:10.1371/journal.pone.0289341)
Supplement: S1 Table — See the section Analysis plan for details on the chose subsets; p refers to the p-value of a X2 test with the null hypothesis of an even distribution of votes, w is the corresponding effect size. These statistics are accompanied by the p-value of an exact binomial test (pexact) and the Bayes Factor estimate for a corresponding Bayesian proportion test computed with the alternative hypothesis in the numerator (BF10). (DOCX) [file pone.0289341.s004.docx]

Table S1.

| Statistic | Main analysis | Full sets only | Name not mentioned | Valence not mentioned |
| --- | --- | --- | --- | --- |
| N | 88 | 84 | 64 | 85 |
| Votes for frequent name | 72 | 69 | 51 | 70 |
| %Frequent | 81.8 | 82.1 | 79.7 | 82.4 |
| Χ²(1) | 35.64 | 34.71 | 22.56 | 35.59 |
| *p* | < .001 | < .001 | < .001 | < .001 |
| W | .636 | .643 | .594 | .647 |
| *p*_exact_ | < .001 | < .001 | < .001 | < .001 |
| *BF*_10_ | 15588908 | 9768348 | 16610 | 15641434 |

Detailed statistics for the validation analyses of Experiment 1, which had employed neutral to positive headlines. See the section Analysis plan for details on the chose subsets; *p* refers to the p-value of a Χ² test with the null hypothesis of an even distribution of votes, w is the corresponding effect size. These statistics are accompanied by the *p*-value of an exact binomial test (*p*_exact_) and the Bayes Factor estimate for a corresponding Bayesian proportion test computed with the alternative hypothesis in the numerator (*BF*_10_).
