# Supplementary material for: Headlines win elections: Mere exposure to fictitious news media alters voting behavior
Source: PLoS One. 2023 Aug 1;18(8):e0289341. doi: 10.1371/journal.pone.0289341 (PMC10393126; doi:10.1371/journal.pone.0289341)
Supplement: S2 Table — See the caption of S1 Table for details on the reported statistics. (DOCX) [file pone.0289341.s005.docx]

Table S2.

| Statistic | Main analysis | Full sets only | Name not mentioned | Valence not mentioned |
| --- | --- | --- | --- | --- |
| N | 89 | 85 | 63 | 67 |
| Votes for frequent name | 37 | 36 | 28 | 29 |
| %Frequent | 41.6 | 42.4 | 44.4 | 43.3 |
| Χ²(1) | 2.53 | 1.99 | 0.78 | 1.21 |
| *p* | .112 | .159 | .378 | .272 |
| w | .169 | .153 | .111 | .134 |
| *p*_exact_ | .137 | .192 | .450 | .328 |
| *BF*_10_ | 0.831 | 0.657 | 0.425 | 0.504 |

Detailed statistics for the validation analyses of Experiment 2 (negative headlines). See the caption of Table S1 for details on the reported statistics.
