# Supplementary material for: Headlines win elections: Mere exposure to fictitious news media alters voting behavior
Source: PLoS One. 2023 Aug 1;18(8):e0289341. doi: 10.1371/journal.pone.0289341 (PMC10393126; doi:10.1371/journal.pone.0289341)
Supplement: S3 Table — See the caption of S1 Table for details on the reported statistics. (DOCX) [file pone.0289341.s006.docx]

Table S3.

| Statistic | Main analysis | Full sets only | Name not mentioned | Valence not mentioned |
| --- | --- | --- | --- | --- |
| N | 144 | 138 | 115 | 141 |
| Votes for frequent name | 91 | 89 | 72 | 91 |
| %Frequent | 63.2 | 64.5 | 62.6 | 64.5 |
| Χ²(1) | 10.03 | 11.59 | 7.31 | 11.92 |
| *p* | .002 | .001 | .007 | .001 |
| w | .264 | .290 | .252 | .291 |
| *p*_exact_ | .001 | < .001 | < .001 | < .001 |
| *BF*_10_ | 25.91 | 56.58 | 7.44 | 66.03 |

Detailed statistics for the validation analyses of Experiment 3 (mixed headlines). See the caption of Table S1 for details on the reported statistics.
