# Supplementary material for: Headlines win elections: Mere exposure to fictitious news media alters voting behavior
Source: PLoS One. 2023 Aug 1;18(8):e0289341. doi: 10.1371/journal.pone.0289341 (PMC10393126; doi:10.1371/journal.pone.0289341)
Supplement: S4 Table — See the caption of S1 Table for details on the reported statistics. (DOCX) [file pone.0289341.s007.docx]

Table S4.

| Statistic | Main analysis | Full sets only | Name not mentioned | Valence not mentioned |
| --- | --- | --- | --- | --- |
| N | 143 | 142 | 110 | 139 |
| Votes for frequent name | 99 | 98 | 75 | 97 |
| %Frequent | 69.2 | 69.0 | 68.2 | 69.8 |
| Χ²(1) | 21.15 | 20.54 | 14.55 | 21.76 |
| *p* | < .001 | < .001 | < .001 | < .001 |
| w | .385 | .380 | .364 | .396 |
| *p*_exact_ | < .001 | < .001 | < .001 | < .001 |
| *BF*_10_ | 6502.50 | 4772.61 | 257.50 | 8916.41 |

Detailed statistics for the validation analyses of Experiment 4 (mixed headlines and a frequency distribution of 100% vs. 0%). See the caption of Table S1 for details on the reported statistics.
